# Evaluation of a Biomedical Informatics course for medical students: a Pre-posttest study at UNAM Faculty of Medicine in Mexico

**DOI:** 10.1186/s12909-015-0349-7

**Published:** 2015-04-01

**Authors:** Melchor Sánchez-Mendiola, Adrián I Martínez-Franco, Marlette Lobato-Valverde, Fabián Fernández-Saldívar, Tania Vives-Varela, Adrián Martínez-González

**Affiliations:** 1 UNAM Faculty of Medicine, Secretariat of Medical Education, Ave. Universidad 3000 C.U., México, D.F. 04510 Mexico; 2 Department of Biomedical Informatics, UNAM Faculty of Medicine, Ave. Universidad 3000, C.U., México, D.F. 04510 Mexico

**Keywords:** Biomedical informatics education, Program evaluation, Undergraduate medical education, Assessment

## Abstract

**Background:**

Biomedical Informatics (BMI) education in medical schools is developing a sound curricular base, but there are few published reports of their educational usefulness. The goal of this paper is to assess knowledge change and satisfaction in medical students after a BMI curriculum.

**Methods:**

The National Autonomous University of México Faculty of Medicine (UNAM) recently implemented a curricular reform that includes two BMI sequential courses (BMI-1 and BMI-2). The research design was one-group pretest-posttest. An objective test with evidence of validity was used for knowledge measurement. A satisfaction questionnaire was applied at the end of the courses. Two-tailed paired Student’s *t-*tests were applied, comparing knowledge scores in the pre and post-test for each course.

**Results:**

The study included student cohorts during two consecutive academic years. The 2013 BMI-1 course (n = 986 students) knowledge pretest score was 43.0 ± 8.6 (mean percent correct ± SD), and the post-test score was 57.7 ± 10.3 (p < 0.001); the 2014 BMI-1 (n = 907) pretest score was 43.7 ± 8.5, and the post-test was 58.1 ± 10.5 (p < 0.001). The 2012 BMI-2 course (n = 683) pretest score was 26.3 ± 7.9, the post-test score was 44.3 ± 13.3 (p < 0.001); the 2013 BMI-2 (n = 926) pretest score was 27.5 ± 7.5, and the post-test was 42.0 ± 11.0 (p < 0.001). The overall opinion of the students regarding the course was from good to excellent, with a response rate higher than 90%. The satisfaction questionnaires had high reliability (Cronbach’s alpha of 0.93).

**Conclusions:**

The study shows a significant increase in BMI knowledge after an educational intervention in four medical student cohorts, and an overall positive evaluation by the students. Long-term follow-up is needed, as well as controlled studies of BMI educational interventions using performance endpoints.

## Background

Healthcare professionals face a variety of challenges in current clinical care systems at a global level, one of which is the appropriate management of data and information [1]. The acquisition and development of competencies in Biomedical Informatics (BMI) are increasingly recognized as fundamental to the effective practice of medicine, and there is a growing movement to include these abilities in the formal curricula of undergraduate and graduate students in the health professions [2-5]. The recent addition by the American Board of Medical Specialties in the United States of Clinical Informatics as a subspecialty has contributed to an explosion of graduate programs in BMI. This formal recognition of the discipline has been a transcendental step in the maturation of BMI as a science and its academic and societal acceptance [6-8].

The increase in opportunities for formal training in BMI at the graduate end of the spectrum of medical education, mainly in Master, PhD and residency programs, has not been accompanied by a similar growth in curricular space for BMI training in medical students [5,9]. The main goal of graduate-level educational programs is to generate professionals with a high level of expertise in the field, but there is a parallel and not sufficiently addressed challenge in the need for dissemination of BMI competencies in healthcare practitioners. Physicians, nurses and allied health sciences personnel require acculturation and professional development in the use of BMI in their fields, and this needs to be addressed both at the undergraduate training and graduate continuing education levels, in order to produce educated users of data, information and knowledge [1,5]. Notwithstanding the appearance of several proposals from the academic community to include BMI competencies in medical schools’ curricula, few published reports actually describe in detail the development and implementation of their BMI programs, and fewer still generate research evidence of their educational impact [2,4,5,10-13].

In developing countries like Mexico, the need for BMI education and implementation is compounded by the fact that technology and information management need to be adapted to the local context. Advances in telemedicine, hospital information systems and electronic health records are dissimilar in different areas of our country (www.cenetec.salud.gob.mx), and the healthcare professionals and trainees that will work and study in these settings need to be aware of the potential beneficial impact of BMI in their practice. We published recently our experience with the development and implementation of BMI courses for medical students in a curricular reform at UNAM Faculty of Medicine in Mexico [9,14], the purpose of this paper is to report evidence of its effectiveness.

## Methods

### Setting

The National Autonomous University of Mexico (UNAM) Faculty of Medicine in Mexico City is the largest medical school in the country and one of the largest in Latin America, with more than 7,000 undergraduate students and more than 8,000 medical residents. It is a public institution and the largest producer of basic and clinical medical research in Mexico, through its affiliations with major national academic medical centers. Recently our MD program underwent a curricular reform that includes two BMI courses (BMI-1 and BMI-2). The new program and the BMI courses are described in detail in our previous papers [9,14].

### Research design and participants

A one-group pre-test post-test quasi-experimental research design was used [15,16]. Pre and post-test knowledge measures were done with an *ad hoc* instrument, at the beginning and end of each BMI-1 and BMI-2 course during two consecutive academic years (2012 to 2014). The sampled population included the student cohorts that were registered at the beginning of each course. We started the study in the second semester of 2012 with the BMI-2 course, continued in 2013 with the BMI-1 and BMI-2 courses, and finished in 2014 with the BMI-1 course, in order to have two cohort rounds of each course (Table 1).

**Table 1:** **Overview of study design and temporal sequence of the courses**

Cohort	Pre-test	BMI courses	Post-test
2012 BMI-2	O	X	O
2013 BMI-1	O	X	O
2013 BMI-2	O	X	O
2014 BMI-1	O	X	O

BMI = Biomedical Informatics.

O = Observation (measurement) of the dependent variable (knowledge test).

X = Exposure to the educational intervention, the independent variable (BMI courses).

### Intervention

The educational interventions were the BMI-1 and BMI-2 courses. The BMI courses are mandatory, one semester long, have 34 curricular hours per course, with a total of 17 two-hour weekly sessions in each program. The BMI-1 course occurs in the second semester of the MD program first year, and the BMI-2 course in the first semester of the second year. The content of the BMI courses was based in a review of the literature, including the major text in the field [1], the International Medical Informatics Association recommendations on education in biomedical and health informatics [17], and some published papers related to the teaching of BMI [4,18]. We developed a BMI textbook in Spanish to provide a local reference and learning resource for the medical students and course teachers, which includes the information and practical activities described in the courses [19].

The programs’ content, teaching methodology and educational objectives are described in detail in our previous paper [9]. Briefly, the goals of the courses are that medical students achieve:

- Competencies in searching, identification and application of biomedical information for the practice of medicine.
- Ability to describe the advances in information and communication technologies relevant to medicine.
- Competencies in effective decision making under conditions of uncertainty.
- Ability to understand and apply current concepts about clinical reasoning and informatics support for clinical decisions.

The courses’ curricula cover BMI themes relevant to the practice of medicine: BMI definition, data-information-knowledge taxonomy, biomedical databases and digital libraries, tools and strategies for information retrieval, hospital information systems, electronic health records, telemedicine, e-learning, ethical and legal aspects of BMI, uncertainty in medicine, cognitive heuristics, Bayes’ theorem, decision analysis, current concepts of clinical reasoning, interpretation of diagnostic tests, cognitive errors, physicians’ and patients’ decision support tools, among others [9].

We used a blended-learning model to take advantage of the online and face-to-face modalities, and developed a virtual learning environment in the Moodle platform. The courses are implemented in the first two years of our medical school program, because in our curriculum we consider BMI as a “basic science” for clinical medicine, and require the students to learn these concepts before they enter the full-time clinical clerkships in the third year [9,14].

### Main outcomes and instrumentation

The independent variables were the BMI courses and the dependent variable BMI knowledge. We also measured attitudes and opinion regarding the programs at the end of each course. For the pre-post test knowledge measurements we developed a multiple-choice question (MCQ) instrument, following Downing’s recommendations for effective test development [20,21]. Items were selected from the courses’ summative exams administered in the initial two years of the program, which had acceptable psychometric characteristics and covered the courses’ content through a test blueprint obtained by consensus. The blueprint and test specifications of the pre-post test were the same as those for the BMI courses’ summative examinations, with the difference that the diagnostic pre-post test had fewer items (the pre-post test had 36 items, and the summative end-of-course exams had 60 items). Our study assessment instrument and the summative exams developed by the BMI Department covered the same themes in equivalent content proportions. The Department of Biomedical Informatics has an Educational Assessment Committee, integrated by six clinician teachers, four informatics professionals and five individuals with formal training in educational assessment, this group developed the tests and collected the exams’ validity and reliability evidence.

In 2012 questions were selected from the item bank, developing a 36-item MCQ exam that covered the BMI-1 course content, and a second exam with the same number of items for the BMI-2 course. Many items were targeted to higher cognitive levels like application and problem solving. The same test was administered the first and last days of each course. The tests were applied through the Moodle online platform used in the BMI courses, and was voluntary. The students had 45 minutes to answer the test, and the results were collected by the BMI Department system administrator in an Excel file that was later transferred to the psychometric analysis software.

Following are a sample of items used in the pre-post test:

- *A hospital hired personnel to manage the database of their patient population. One employee sold the database to a healthcare products company. Which of the following informatics ethics principles were violated?*

- *Access and legitimate infringement*
- *Openness and access*
- *Security and privacy*
  *The main difference between Bioinformatics and Medical Informatics is that the first focuses on the following area:*
- *Biology*
- *Biomolecular*
- *Biotechnological*
  *When do “data” become “information” in medicine?*
- *When they are incorporated in our memory*
- *When they acquire meaning*
- *When they are applied in practice*
  *Which of the following MEDLINE search strategies is most likely to retrieve the largest number of references?*
- *bacterial meningitis AND dexamethasone*
- *bacterial meningitis NOT dexamethasone*
- *bacterial meningitis OR dexamethasone*
  *When we say that a patient has a “textbook presentation” of a disease, and on that basis we estimate our diagnostic hypotheses, which of the following heuristics are we predominantly using?*
- *Anchoring and adjustment*
- *Availability*
- *Representativeness*
  *An intern sees a patient in the clinic with pregnancy-induced hypertensive disorder, and has not had time to review the medical literature where some important evidence about a therapeutic modality has recent been published. What source of uncertainty is most likely to occur in this situation?*
- *Conceptual*
- *Personal*
- *Technical*

We also applied a program evaluation survey to the students at the end of each course, a 41-item questionnaire that explored several aspects of the programs. The students answered the course evaluation instrument in the Moodle platform after the last session, at the same time they filled teachers’ evaluation questionnaires. After the information was collected, it was summarized without identifiers by personnel not directly involved in the study. The instrument had been used previously in our courses, with a high reliability (Cronbach’s alpha = 0.93) [9].

### Supplementary sources of BMI learning evidence

Furthermore, there were two partial summative exams in each course, as components of the formal curriculum assessment activities, with two components: a 60-item multiple-choice question test, and a practical hands-on test in the computer lab that explored competencies such as use of Medline, appraisal of the basic elements of a telemedicine consultation case, among others. At the end of each course the students had a final summative exam.

### Statistical analysis

Only the data from students that responded the pre and post-tests were included. Two-tailed paired Student’s *t-*test was applied, comparing knowledge scores in the pre and post-test for each course, using PRISM version 6 for Macintosh (http://www.graphpad.com/scientific-software/prism/). P-values less than 0.05 were considered statistically significant. Item analysis of the MCQ tests was performed with ITEMAN for Windows version 4, (Assessment Systems Corporation, St. Paul, MN www.assess.com). Cohen’s *d* with pooled standard deviations was calculated as a measure of effect size for the knowledge scores’ changes among groups, using the following formula [16,22]:

$$ \mathrm{Cohen}\hbox{'}\mathrm{s}\kern0.24em d={M}_1-{M}_2/{\sigma}_{\mathrm{pooled}} $$

Where: M = mean of each group

σ *=* standard deviation

σ_pooled_ = √[(σ _1_^2^+ σ _2_^2^) / 2]

### Ethical aspects

The study was in compliance with the Declaration of Helsinki of ethical principles for research involving human subjects. The assessment of the BMI courses was approved by the Faculty of Medicine Technical Council and Curriculum Committee, as part of the new curriculum evaluation. There was no individual written informed consent, since the study was done as part of the program evaluation and quality improvement process, the tests were voluntary and the data are described in aggregate and anonymous fashion.

## Results

A total of 3,502 students completed both pre and post-tests for all BMI-1 and BMI-2 courses. The student distribution per course is shown on Table 2.

**Table 2:** **Number of students that received the BMI courses and pre-post tests**

	2012 BMI-2	2013 BMI-1	2013 BMI-2	2014 BMI-1
Total of registered students	1169	1355	1037	1324
Students that completed pre and post-tests	683 (58.4%)	986 (72.8%)	926 (89.3%)	907 (68.5%)

The gender distribution of the sampled population was 62.7% female, 37.3% male, mirroring the sex percentages of the total undergraduate medical school population in our institution.

The psychometric analysis for the diagnostic pre-post test and the BMI Department summative examinations was performed with the software Iteman, which uses the Classical Measurement Theory (CMT) conceptual framework. The reliability of our pre-post tests measured with Cronbach’s alpha ranged from 0.5 to 0.7, and the reliability of the summative exams from 0.80 to 0.90. The lower reliability of the pre-post test is acceptable and reasonable for a non-summative diagnostic test. The p-value (difficulty index in psychometric parlance, which represents the percentage of items answered correctly in the test; the higher the p-value the easier the test) of the diagnostic post-test was about 0.58 in the BMI-1 courses and 0.42-0.44 in the BMI-2 courses, whereas the difficulty index of the summative tests fluctuated around 0.67. The mean point biserial correlation index (a discrimination index that describes the relationship between a student’s response to a question and the total score on the test; it is useful to differentiate among students in terms of ability) of the diagnostic post-test was 0.2, and the discrimination index of the summative exams ranged from 0.24 to 0.38.

The mean percent correct scores of the pre and post-test for each BMI course are shown in Table 3.

**Table 3:** **Results of pre and post-tests knowledge levels for each course**

	2012 BMI-2		2013 BMI-1		2013 BMI-2		2014 BMI-1	
	PRE	POST	PRE	POST	PRE	POST	PRE	POST
Number of students	683	683	986	986	926	926	907	907
Mean % correct score ± SD	26.3 ± 7.9	44.3 ± 13.3*	43.0 ± 8.6	57.7 ± 10.3*	27.5 ± 7.5	42.0 ± 11.0*	43.7 ± 8.5	58.1 ± 10.5*
95% Confidence interval	25.7, 26.9	43.3, 45.3	42.5, 43.6	57.1, 58.4	27.0, 28.0	41.3, 42.7	43.2, 44.3	57.4, 58.8

* *p* < 0.001, pre vs. post comparison with paired Student’s *t*-test.

The increase in knowledge effect size at the end of the courses, measured with Cohen’s *d*, was 1.56 for the 2013 BMI-1 course; 1.50 for the 2014 BMI-1 course; 1.66 for the 2012 BMI-2 course; and 1.55 for the 2013 BMI-2 course.

Since we included the totality of our available student population, and due to the large size of our student cohorts, we deemed not necessary to do an *a priori* sample size calculation. We performed *post-hoc* power calculations and for our comparisons the power was >95%, which means that the possibility of a type 2 or beta error is extremely unlikely. This is reasonable since our simple sizes are large and the pre-post differences are considerable.

**Figure 1:**
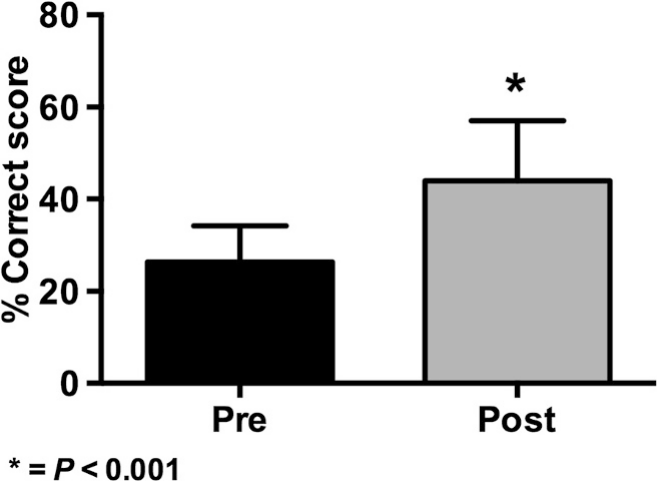
Results of knowledge scores (mean ± SD) in pre and post-tests for the 2012 BMI-2 course (* = p < 0.001 with paired Student’s ***t*** **-test; n = 683).**

**Figure 2:**
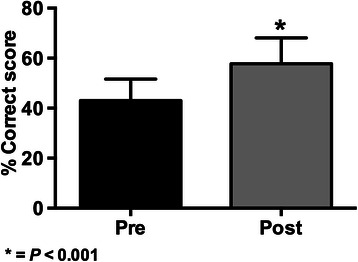
Results of knowledge scores (mean ± SD) in pre and post-tests for the 2013 BMI-1 course (* = p < 0.001 with paired Student’s ***t*** **-test; n = 986).**

**Figure 3:**
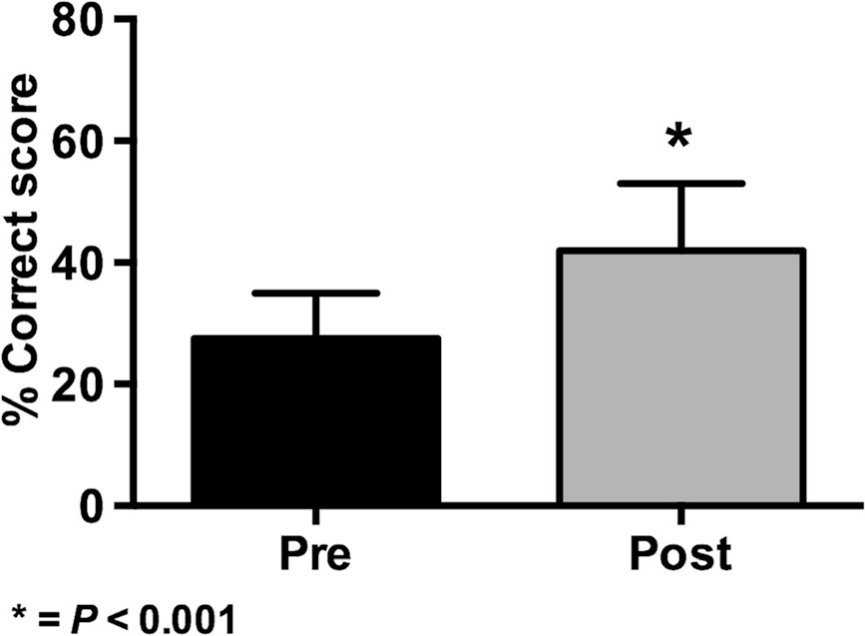
Results of knowledge scores (mean ± SD) in pre and post-tests for the 2013 BMI-2 course (* = p < 0.001 with paired Student’s ***t*** **-test; n = 926).**

**Figure 4:**
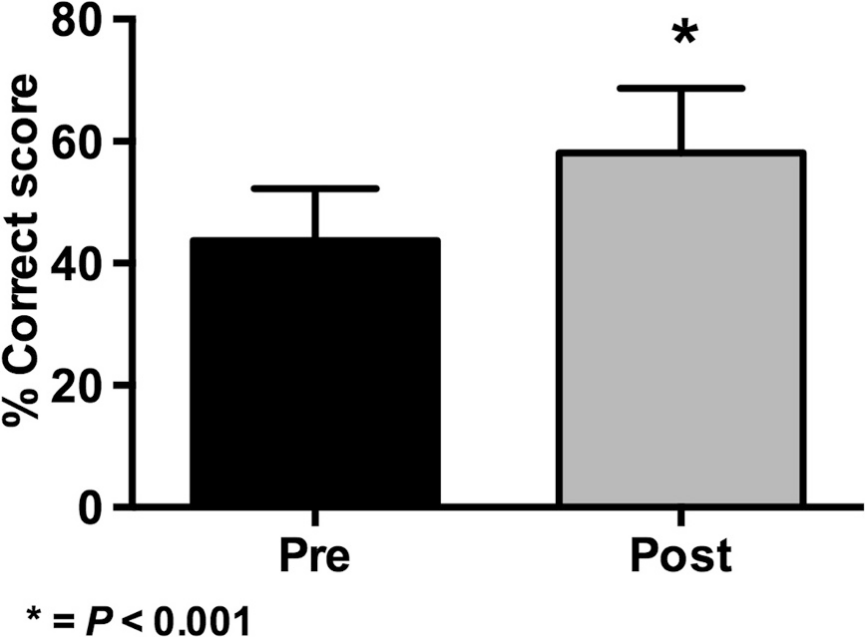
Results of knowledge scores (mean ± SD) in pre and post-tests for the 2014 BMI-1 course (* = p < 0.001 with paired Student’s ***t*** **-test; n = 907).**

The graphical comparison of the pre and post-test knowledge exams results are shown in Figures 1, 2, 3 and 4.

Regarding the supplementary evidence obtained in the courses’ curricular summative evaluations, the 60-item MCQ exams had an average Cronbach's alpha of 0.83, a mean difficulty index p-value of 0.66 and a mean point-biserial correlation index of 0.29. At the end of all the BMI courses 93.6% of the student population had passing scores. The average final grade in all groups obtained by averaging the curricular summative exams’ scores with the teacher’s grades was 7.8 (in the scale of 0 to 10 used in the Mexican educational system), with 57.7% of the students achieving a final grade of 8 or above.

The data from the curricular summative exams and the teachers’ grades, which are supplementary evidence of learning in the BMI courses, are shown in Table 4.

**Table 4:** **Results of the courses’ curricular summative exams and teachers’ grades (grades are in a scale of 0 to 10)**

	Teachers’ grade (mean ± SD)	Exams (mean ± SD)	Final grade (mean ± SD)	% Accredited the course
**2012 BMI-2**	9.3 ± 1.1	7.0 ± 1.1	8.0 ± 1.1	94.3
**2013 BMI-I**	8.1 ± 2.3	6.6 ± 1.1	7.4 ± 1.1	89.5
**2013 BMI-2**	9.2 ± 1.1	7.2 ± 1.2	8.2 ± 0.9	96.9
**2014 BMI-I**	8.4 ± 2.7	6.2 ± 2.2	7.3 ± 2.2	94.5
**All courses**	8.7 ± 0.5	6.7 ± 0.4	7.7 ± 0.4	93.8

The program evaluation anonymous survey had a Cronbach’s alpha of 0.93, and a high return rate (>90% of the student population in all the courses). The overall opinion of the students regarding the different elements of the program was excellent, with 88% to 97% of the responses in the “adequate” or “very adequate” side of the four-level Likert scale. The student evaluations were similar in the dimensions of infrastructure, educational activities, course resources and perception of clinical relevance, the detailed results are shown in Tables 5 and 6.

**Table 5:** **Student evaluation of the Biomedical Informatics-1 courses (2013 and 2014) at UNAM Faculty of Medicine (n = 2098)**

Questionnaire items	Student answers									
	Very Inadequate		Inadequate		Adequate		Very adequate		No answer	
	#	%	#	%	#	%	#	%	#	%
The functioning of the computer equipment was	7	0.3	47	2.2	787	37.5	1235	58.9	22	1.0
The functioning of the virtual classroom was	11	0.5	74	3.5	769	36.7	1218	58.1	26	1.2
The software programs used were	5	0.2	63	3.0	819	39.0	1189	56.7	22	1.0
The design of the course sessions was	14	0.7	99	4.7	948	45.2	1001	47.7	36	1.7
The audiovisual teaching material was	14	0.7	119	5.7	836	39.8	1095	52.2	34	1.6
The online learning exercises were	47	2.2	202	9.6	1008	48.0	806	38.4	35	1.7
The clinical cases used were	31	1.5	212	10.1	1046	49.9	781	37.2	28	1.3
The number of participants in my subgroup was	32	1.5	161	7.7	929	44.3	939	44.8	37	1.8
The team performance of my subgroup was	72	3.4	240	11.4	909	43.3	840	40.0	37	1.8
The bibliographic material was	25	1.2	120	5.7	880	41.9	1041	49.6	32	1.5
Having two teachers per group (physician and informatician) was	32	1.5	67	3.2	735	35.0	1236	58.9	28	1.3
	**Never**		**Sometimes**		**Usually**		**Always**		**No answer**	
	#	%	#	%	#	%	#	%	#	%
Teachers made me reflect on how to apply this knowledge in my professional life	23	1.1	107	5.1	772	36.8	1167	55.6	29	1.4
Critical appraisal of the teaching material was promoted by the teachers	28	1.3	125	6.0	962	45.9	945	45.0	38	1.8
The course made me reflect on its relationship with my other courses	54	2.6	205	9.8	900	42.9	910	43.4	29	1.4
Teachers promoted study time out of the school	67	3.2	298	14.2	1004	47.9	692	33.0	37	1.8
The course encouraged me to seek more information on the subject	84	4.0	255	12.2	902	43.0	824	39.3	33	1.6
Teachers gave me feedback on my verbal and written communication skills	91	4.3	221	10.5	881	42.0	871	41.5	34	1.6
Clinical medical terminology was incorporated in the course	39	1.9	153	7.3	904	43.1	965	46.0	37	1.8
The assignment papers were returned with feedback	89	4.2	213	10.2	817	38.9	945	45.0	34	1.6
The course promoted the development of skills and abilities	39	1.9	103	4.9	867	41.3	1046	49.9	43	2.0
Clinical cases were used in class to learn the themes	21	1.0	102	4.9	887	42.3	1050	50.0	38	1.8
Teachers considered physician-patient relationship issues in class	9	0.4	53	2.5	724	34.5	1273	60.7	39	1.9
Teachers pointed the importance of ethical issues in dealing with patients	8	0.4	34	1.6	660	31.5	1362	64.9	34	1.6
Teachers pointed the importance of preventive actions in the clinical cases	13	0.6	58	2.8	793	37.8	1195	57.0	39	1.9
Teachers noted the epidemiology of the problems discussed in the course	18	0.9	90	4.3	903	43.0	1050	50.0	37	1.8
Teachers promoted the development of professional values in the course	16	0.8	63	3.0	843	40.2	1137	54.2	39	1.9
	**Very easy**		**Easy**		**Difficult**		**Very difficult**		**No answer**	
	#	%	#	%	#	%	#	%	#	%
The difficulty level of the course was	41	2.0	157	7.5	1146	54.6	729	34.7	25	1.2
	**Unsatisfied**		**Little satisfied**		**Satisfied**		**Very satisfied**		**No answer**	
	#	%	#	%	#	%	#	%	#	%
My expectations of the course were	74	3.5	235	11.2	968	46.1	792	37.8	29	1.4
	**Insufficient**		**Sufficient**		**Good**		**Excellent**		**No answer**	
	#	%	#	%	#	%	#	%	#	%
The knowledge and skills I acquired on the course were	33	1.6	164	7.8	1000	47.7	870	41.5	31	1.5

**Table 6:** **Student evaluation of the Biomedical Informatics-2 courses (2012 and 2013) at UNAM Faculty of Medicine (n = 2122)**

	Very Inadequate		Inadequate		Adequate		Very adequate		No answer	
	#	%	#	%	#	%	#	%	#	%
The functioning of the computer equipment was	10	0.5	26	1.2	712	33.6	1293	60.9	81	3.8
The functioning of the virtual classroom was	15	0.7	33	1.6	719	33.9	1272	59.9	83	3.9
The software programs used were	12	0.6	48	2.3	830	39.1	1145	54.0	87	4.1
The design of the course sessions was	23	1.1	154	7.3	999	47.1	858	40.4	88	4.1
The audiovisual teaching material was	32	1.5	169	8.0	947	44.6	887	41.8	87	4.1
The online learning exercises were	48	2.3	236	11.1	993	46.8	759	35.8	86	4.1
The clinical cases used were	41	1.9	244	11.5	995	46.9	753	35.5	89	4.2
The number of participants in my subgroup was	51	2.4	207	9.8	1014	47.8	761	35.9	89	4.2
The team performance of my subgroup was	63	3.0	237	11.2	979	46.1	754	35.5	89	4.2
The bibliographic material was	62	2.9	210	9.9	958	45.1	801	37.7	91	4.3
Having two teachers per group (physician and informatician) was	46	2.2	152	7.2	703	33.1	1136	53.5	85	4.0
The inclusion of the program DXplain in the course was	78	3.7	257	12.1	945	44.5	752	35.4	90	4.2
The DXplain interface was	58	2.7	176	8.3	740	34.9	1061	50.0	87	4.1
DXplain functions and operation were	19	0.9	70	3.3	616	29.0	1330	62.7	87	4.1
The knowledge and skills acquired after using DXplain were	16	0.8	92	4.3	690	32.5	1231	58.0	93	4.4
The application of DXplain in other courses was	20	0.9	110	5.2	702	33.1	1196	56.4	94	4.4
The decision making in the clinical cases using DXplain was	43	2.0	164	7.7	748	35.2	1070	50.4	97	4.6
The use of DXplain for achieving the objectives of the course was	35	1.6	192	9.0	804	37.9	996	46.9	95	4.5
The time dedicated in class for using DXplain was	25	1.2	111	5.2	829	39.1	1060	50.0	97	4.6
The exercises used in the course with DXplain were	27	1.3	121	5.7	840	39.6	1044	49.2	90	4.2
The knowledge of the teacher in the use of DXplain was	23	1.1	132	6.2	876	41.3	998	47.0	93	4.4
The strategies used by the teacher for teaching DXplain were	17	0.8	92	4.3	757	35.7	1161	54.7	95	4.5
How do you consider the inclusion of DXplain for medical students	20	0.9	76	3.6	626	29.5	1310	61.7	90	4.2
	**Never**		**Sometimes**		**Usually**		**Always**		**No answer**	
	#	%	#	%	#	%	#	%	#	%
The assignment papers were returned with feedback	116	5.5	308	14.5	978	46.1	625	29.5	95	4.5
The course made me reflect on its relationship with my other courses	82	3.9	268	12.6	990	46.7	693	32.7	89	4.2
	**Very easy**		**Easy**		**Difficult**		**Very difficult**		**No answer**	
	#	%	#	%	#	%	#	%	#	%
The difficulty level of the course was	61	2.9	165	7.8	863	40.7	950	44.8	83.0	3.9
	**Unsatisfied**		**Little satisfied**		**Satisfied**		**Very satisfied**		**No answer**	
	#	%	#	%	#	%	#	%	#	%
My expectations of the course were	105	4.9	235	11.1	955	45.0	741	34.9	86	4.1
	**Insufficient**		**Sufficient**		**Good**		**Excellent**		**No answer**	
	#	%	#	%	#	%	#	%	#	%
The knowledge and skills acquired on the course were	45	2.1	232	10.9	1038	48.9	715	33.7	92	4.3

## Discussion

There are few published reports of BMI curricula in medical schools, most are program descriptions, their development, contents and teaching methods. A few are narratives of the status of BMI education in their countries, descriptive observational reviews of the programs’ characteristics, university affiliation and contents, but as far as we could ascertain none report research data about their educational effectiveness [18,23-33]. Our study measured knowledge acquisition of BMI in undergraduate medical students, and demonstrated a substantial increase in knowledge after the educational experiences and a positive opinion about the courses.

In a recent paper, Silverman and colleagues reported the design, implementation and evaluation of a BMI course for medical students at the Arizona College of Medicine campus in Phoenix, USA [13]. Their curricular model is more integrated than ours, which allows for better longitudinal coordination and integration at a higher level. Their medical school has been implementing BMI education for a longer period, since 2005, and has evolved to a coherent educational intervention through several modifications. One of the more challenging tasks in our medical school during the implementation and evaluation of the BMI curriculum, is the size of our student and faculty body. The Arizona College of Medicine medical school has a student body of less than 600, and each year they graduate about 115 students (http://en.wikipedia.org/wiki/University_of_Arizona_College_of_Medicine), which allows for more dynamic and relatively short-term curricular changes, as well as faculty development initiatives. UNAM Faculty of Medicine has more than 7000 undergraduate students and more than 2000 faculty, so our organizational structure increases the complexity of curricular innovations implementation. Silverman used several subjective and objective measures of program evaluation (questionnaires, pre-post self-assessment instruments), and demonstrated improvements in the medical students’ self-assessment scores between pre and post-course, which persisted during the third year of medical school [13]. The instruments they used were opinion questionnaires and self-assessed estimates of knowledge, which are not as indicative of learning as external objective instruments, similar to the ones used in our study. Self-assessment is a complex and difficult topic in medical education, but the majority of evidence suggests that physicians and clinicians in training are poor at self-assessment [34,35]. Their self-reported assessment of knowledge used a Likert scale of agreement with several statements related to BMI abilities, which is not directly comparable with our estimates of knowledge that used an objective test with percent correct scores. Their student evaluations are optional, so their response rates were relatively low, in 2009 they had 34% of responses from a class of 47 students, and 38% in a class of 45 students. In our setting we had a higher response rate in our student evaluations, probably due to the mandatory nature of course and teacher evaluation in our institution. In summary, the direction of change in BMI knowledge was positive in both studies, although the magnitude of this change cannot be directly compared due to the different scales of the measurement instruments. Overall, the nature of the obstacles and barriers to their BMI curricular innovation appear to be similar to ours, and require comprehensive longitudinal evaluation and intense integration efforts.

We didn’t find published papers that used an objective measure of BMI knowledge achievement as indicator of BMI interventions’ educational effectiveness in medical schools, our paper appears to be the first report that shows a significant increase in BMI knowledge in medical students. There are difficulties in searching published research studies of BMI educational interventions, because there are papers that include the term “informatics” in their titles or abstracts, but really are focused on evidence-based medicine, information retrieval or library skills (e.g. the paper by Badgett et al., titled “Teaching clinical informatics to third-year medical students: negative results from two controlled trials”, is actually about the use of Medline for evidence retrieval, not BMI in its current sense) [36]. After using several search strategies in many databases we were not able to find other research papers that measured BMI knowledge acquisition with instruments that had evidence of validity.

Our BMI-1 and BMI-2 courses are associated with a large and statistically significant increase in knowledge, and overall had a positive evaluation by the students. The consistency of the knowledge increase in the consecutive courses adds validity to our findings, the pre and immediate post measures are almost identical in the two consecutive student cohorts for each BMI course, suggesting a similar baseline level of knowledge and an equivalent amount of knowledge acquisition. In social sciences usually the larger the effect size, the greater the impact of an intervention. Cohen suggested that an effect size of 0.8 is large, 0.5 is moderate, and 0.2 is small [16]. In our study all effect sizes are above 1.5, which is large and probably educationally significant. It is important to note that the study instruments were applied in a voluntary non-summative fashion, so the students may not have applied the same effort to answer them correctly as for summative tests. This phenomenon is apparent when we compare the percent correct scores of our study pre-post diagnostic tests with the actual scores of the BMI 1 and 2 courses’ curricular summative exams, which had scores above 70% compared to the 40-60% scores found in our study. Students tend to have higher scores in summative exams than in formative diagnostic voluntary tests [37]. Nonetheless, our finding of a significant increase in knowledge with the same instrument in several consecutive cohorts of students lends reproducibility and validity to our results.

Cook and Bordage recently proposed a classification of medical education research studies based on their purpose: description studies (what was done?), justification studies (did it work?) and clarification studies (how or why did it work?), and found in a selected review of the medical education literature that 72% were justification studies, 16% descriptive and 12% clarification studies [38]. Research performed to document if educational interventions work is justified, because many teaching strategies and courses are implemented in medical schools and hospitals without any evidence of their effectiveness, and all require resources to be applied and implemented. BMI educational interventions in particular, as shown in the description of our courses’ design and implementation, require a substantial amount of financial, human and technological resources to be effectively implemented [9], so in the current educational and healthcare arenas of accountability and resource limitations, it is relevant to identify if educational interventions work.

Some authors suggest that studies that demonstrate “if you teach them they will learn” are not very relevant, since medical students are by definition high-achieving individuals with strong academic credentials, so if you teach them something (and the content is mandatory and appears in exams) they will dedicate substantial effort to learn it and do well on the tests [39]. We argue that it’s important to add to the published literature objective evidence that teaching BMI is associated with increase in knowledge, mainly because the construct of BMI is fuzzy and a moving target, and this information is relevant enough to justify its inclusion in curricular reforms.

The study has the following limitations: randomized controlled trials with strict experimental design are the best way to demonstrate the effectiveness of an intervention, but ethical and logistical issues inherent to the realities of medical schools’ activities made it impossible for us to use this design; the one-group pre-post test study design, with no control group, makes the study prone to internal validity threats (history, maturation, instrument decay, testing, among other). We recognize these potential threats but made our best efforts to control them to the extent possible within the confines of the study design; our main outcomes were knowledge and satisfaction, and knowledge was measured only with MCQ tests, which are limited for assessing competence and performance, however the study participants are novice medical students with no formal clinical responsibilities, so knowledge improvement was a more realistic goal; the study reflects only one school and our particular curriculum, so the external validity of the findings to other institutions with different programs could be questionable, nonetheless our curriculum has a solid design with sound educational strategies, and the consistency in our findings with different cohorts of students argues for the validity to our conclusions. We plan to design a study that evaluates different medical schools, to address some of the issues discussed in our study.

The pre and post-test instruments were the same, as mentioned in Methods. In one-group pre-posttest quasiexperimental research the following paradoxical dilemma occurs: if you use a different test for the pre and post, you have to demonstrate unequivocally that the tests are similar in difficulty, otherwise the inference of difference in achievement between groups cannot be done; and if you use the same test, as we did in our study, the “testing threat” to internal validity can introduce bias in the measurement [16]. This risk of bias cannot be completely excluded in quasiexperimental design. We argue, however, that our sample sizes, the magnitude of the differences, the diagnostic nature of our tests, and the time interval between pre and post-test (one semester) can contribute to attenuate this potential bias.

The course satisfaction questionnaire could also have some response bias (social desirability, fear of retaliation if too critical), which we attempted to diminish by using best practices in questionnaire design, administration and analysis, including the anonymization of students’ responses. We acknowledge that the high reliability in our study satisfaction questionnaire does not exclude response bias and threats to validity.

How early should BMI be taught in the medical curriculum? Shortliffe maintains that BMI training should start at the undergraduate level in medical school and the healthcare professions [4,5], when the professional identity of physicians, nurses and other practitioners is being developed. Ideally BMI should be incorporated in an integrated and longitudinal fashion throughout the curriculum to achieve optimal educational outcomes, this is nonetheless not always feasible. In our setting, with a traditional curriculum of basic sciences the first two years and three years of clinical rotations, it made more sense both educationally and logistically to initiate the BMI courses with the other “basic” sciences, as a fundamental building block necessary for the remainder of the clinical phase of medical training. There are advantages and disadvantages to this approach, and each medical school should approach the curricular design task carefully, taking into account their educational model and available resources, making explicit efforts to integrate BMI competencies during clinical training.

There is controversy in the medical education literature about the different levels of outcomes that should be sought in educational interventions and research studies. Kirkpatrick’s evaluation framework has been traditionally used for medical education and in some essays of BMI educational interventions [40,41]. The lower levels of this framework are satisfaction and knowledge acquisition, and the higher levels (more important and relevant to society) are behavioral changes of physicians, improvement in clinical outcomes in patients and in the community. These arguments are relevant, but the model has been criticized recently. Educational interventions that occur early in medical school training cannot be held to the same standards as interventions that occur when a doctor is already practicing or is involved in continuing medical education activities, the time gap is too large and there are too many confounding factors and intervening variables [42]. Since our students in the first two years still do not have direct responsibility with patients, we think that knowledge acquisition and satisfaction are more realistic and reasonable endpoints. On the other hand, there is a substantial amount of literature that suggests that increases in knowledge and test scores in MCQ exams have a positive and statistically significant correlation with competence and performance. Knowledge and performance aren’t that separable, since it takes knowledge to perform. Typical correlations between measures of knowledge and performance are in the range of 0.6 to 0.9 [43].

The increasing sophistication of technology and educational strategies predict a fascinating scenario where they can interact and make the learning of BMI much more integral to clinical practice, as Otero and Hersh suggest in a recent Web 3.0 and Education 3.0 essay [44]. Our institution has recently started a project called “All UNAM online”, with emphasis on sharing educational material through the web. We are currently in the process of adapting the BMI courses educational materials for online distribution, where they will be available in the near future (http://www.unamenlinea.unam.mx).

Finally, the current emphasis on evidence-based medicine, healthcare learning organizations, patient safety and quality of care, provide an appropriate scenario for advancing the importance of teaching and learning BMI in health professions schools and academic health centers [1,45]. The Association of American Medical Colleges and the Howard Hughes Medical Institute recently defined scientific competencies for future medical school graduates, and one of these competencies is: “*Apply quantitative knowledge and reasoning—including integration of data, modeling, computation, and analysis—and informatics tools to diagnostic and therapeutic clinical decision making*” [46].

## Conclusions

Our study shows a significant increase in BMI knowledge after an educational intervention in four medical student cohorts, and an overall positive evaluation by the students. Long-term follow-up is needed, as well as controlled studies of BMI educational interventions using performance endpoints.

## Abbreviations

BMI: Biomedical informatics
MCQ: Multiple choice questions
UNAM: National Autonomous University of México
